# FeS_2_/CuFeS_2_ Composite Anodes Based on Seafloor Massive Sulfides Compositions for Lithium-Ion Batteries

**DOI:** 10.3390/ma19112199

**Published:** 2026-05-23

**Authors:** Songkai Yan, Xuefeng Yin, Moxuan Chen, Ouyuan Lu, Chunyu Chen, Dianchun Ju

**Affiliations:** School of Metallurgy Engineering, Jiangsu University of Science and Technology, Zhangjiagang 215600, China; yansongkai0626@163.com (S.Y.); 18862658620@163.com (X.Y.); 17813025840@163.com (M.C.); 13375293818@163.com (O.L.); chen_chunyu@just.edu.cn (C.C.)

**Keywords:** seafloor massive sulfides, pyrite, chalcopyrite, anode materials, mineral-inspired design

## Abstract

Transition metal sulfides are promising anode materials for lithium-ion batteries, but their practical application is limited by severe volume variation and sluggish reaction kinetics during cycling. Inspired by the natural mineral assemblage of seafloor massive sulfides (SMS), FeS_2_/CuFeS_2_ composite anodes were prepared by a mechanochemical ball-milling method with mass ratios of 9:1 and 7:3 to reflect the major compositional characteristics of SMS. Among them, the 9:1 composite (F9C1) exhibited the best overall electrochemical performance, delivering a reversible capacity of 763.4 mAh g^−1^ after 300 cycles at 1 C and retaining 46% of its baseline capacity at 5 C. Structural and electrochemical analyses suggested that the introduction of a small amount of CuFeS_2_ likely promoted interfacial interactions between FeS_2_ and CuFeS_2_ phases, reduced charge-transfer resistance, and enhanced pseudocapacitive contribution, while preserving the capacity advantage of the FeS_2_ host phase. These results demonstrate that mineral-inspired compositional design is an effective strategy for improving the lithium-storage performance of sulfide anodes and provides a feasible route for developing electrode materials inspired by naturally coexisting sulfide minerals.

## 1. Introduction

The ongoing transition toward a low-carbon energy system has driven unprecedented growth in clean energy technologies, particularly electric vehicles (EVs) and large-scale energy storage systems (ESS) [1,2]. In the field of lithium-ion batteries, the limited theoretical capacity of commercial graphite anodes (~372 mAh g^−1^) has become a critical bottleneck for further increasing energy density [2,3]. Consequently, the development of alternative anode materials with higher capacity and improved cycling stability is of paramount importance [4].

Transition metal sulfides (TMSs) have emerged as promising candidates owing to their high theoretical capacities and diverse electrochemical reaction mechanisms [5,6]. Among them, pyrite (FeS2) has attracted considerable interest due to its high theoretical capacity (894 mAh g^−1^), natural abundance, and low cost [2,7]. However, its practical application is severely hindered by intrinsically low electrical conductivity and drastic volume expansion during cycling, which lead to rapid capacity fading [7,8,9].

To overcome these limitations, various strategies have been explored, including carbon coating and multiphase composite construction. For instance, FeS_2_@C composites have been shown to improve rate capability, while FeS_2_-based composite systems such as FeS_2_/CoS and FeS_2_/CoS/C exhibit enhanced reversible capacity and cycling stability [10,11]. Meanwhile, bimetallic sulfides offer additional advantages over single-metal sulfides through multicomponent synergy, which can alleviate volume variation and provide more favorable electrochemical kinetics, often with higher charge/discharge voltage plateaus [12,13]. More importantly, the construction of multiphase sulfide composites has been widely recognized as an effective strategy for mitigating the intrinsic limitations of single-metal sulfides. In such systems, phase interactions introduced by compositing can shorten solid-state diffusion pathways and accelerate redox kinetics, thereby leading to improved electrochemical performance [14,15].

Chalcopyrite (CuFeS_2_), as a representative bimetallic sulfide, combines these merits with the additional advantage of relatively high electrical conductivity and low raw-material cost [2,16,17]. Zhang et al. [18] showed that fine CuFeS_2_ powders derived from natural chalcopyrite could deliver high reversible capacities even after extended cycling, highlighting the importance of particle-size regulation for promoting ion transport and electrochemical performance [15,19]. Li et al. [20] further demonstrated that coupling CuFeS_2_ with expanded graphite can effectively improve electrochemical behavior by enhancing charge transport and suppressing polysulfide shuttling. Nevertheless, previous studies have primarily focused on external conductive modification or conventional binary compositing strategies, while the influence of naturally inspired compositional ratios on sulfide phase synergy remains insufficiently understood.

Recent studies have demonstrated that FeS_2_-based sulfide anodes can deliver high lithium-storage capacities through conversion reactions; however, simultaneously achieving high reversible capacity, long-term cycling stability, and acceptable initial Coulombic efficiency remains challenging. As summarized in Table 1, most reported FeS_2_/carbon composites and related bimetallic sulfide systems typically exhibit reversible capacities of ~480–710 mAh g^−1^ after 100–300 cycles, often accompanied by considerable irreversible lithium consumption during the initial cycles [21,22]. Although strategies such as carbon modification, heterostructure engineering, and multicomponent coupling can improve conductivity and structural stability, excessive phase complexity may also compromise the intrinsic capacity contribution of FeS_2_. Therefore, developing a compositionally optimized sulfide system capable of balancing high reversible capacity, structural stability, and electrochemical reversibility remains an important challenge for conversion-type sulfide anodes.

Seafloor massive sulfides (SMS) are naturally occurring polymetallic sulfide deposits composed of multiple coexisting sulfide minerals, including Fe-containing and Cu-containing sulfide phases [23,24,25,26]. The naturally intergrown multiphase characteristics of SMS provide a useful compositional reference for designing multiphase sulfide electrode materials. In our previous work, SMS ores were directly utilized as lithium-ion battery anode materials through a mechanochemical ball-milling strategy, where different milling durations significantly influenced the electrochemical behavior of the materials. Further phase analysis of a representative SMS sample (TVG32, China Ocean Sample Repository) revealed that pyrite (FeS_2_), chalcopyrite (CuFeS_2_), and sphalerite (ZnS) were the dominant sulfide phases, among which the compositional ratio between FeS_2_ and CuFeS_2_ was close to 9:1.

Inspired by this naturally occurring mineral assemblage, commercially sourced high-purity natural pyrite (FeS_2_) and chalcopyrite (CuFeS_2_) minerals (>99.99%) were employed in the present work to construct model FeS_2_/CuFeS_2_ composite systems with controlled mass ratios (9:1 and 7:3) via a mechanochemical approach. The 7:3 sample was introduced as a comparison group to further investigate the influence of phase proportion on electrochemical behavior. Compared with the complex multiphase composition of natural SMS ores, the simplified FeS_2_/CuFeS_2_ model system enables a clearer investigation of the synergistic effects between the dominant sulfide phases while minimizing interference from gangue minerals and secondary components. Mechanochemical synthesis is widely regarded as a promising strategy for material fabrication due to its simplicity, environmental friendliness, and scalability [27,28,29]. Through ball-milling treatment, not only can homogeneous mixing of different components be achieved, but lattice distortion and partial amorphization may also be induced, which are beneficial for enhancing electrochemical performance [30,31].

We hypothesize that introducing a minor CuFeS_2_ phase into the FeS_2_ matrix can regulate charge-transfer kinetics and phase-interaction effects while preserving the high-capacity characteristics of the FeS_2_ host phase. In contrast, excessive CuFeS_2_ content may dilute the capacity contribution of FeS_2_ and weaken the overall energy-storage capability. Therefore, a compositional ratio close to that of naturally occurring mineral assemblages may provide an optimized balance between electrochemical activity and structural stability. Inspired by the compositional characteristics of naturally coexisting sulfide minerals in SMS systems, this study systematically investigates the synergistic electrochemical effects between FeS_2_ and CuFeS_2_ phases and clarifies the influence of compositional ratio on lithium-storage behavior. The findings of this work are expected to provide insights into compositionally inspired sulfide-electrode design and offer a feasible strategy for developing low-cost multiphase sulfide anode materials.

## 2. Materials and Methods

### 2.1. Materials and Reagents

Pyrite (FeS_2_, analytical grade) and chalcopyrite (CuFeS_2_, analytical grade) were purchased from Aladdin Biochemical Technology Co., Ltd. (Shanghai, China) and used without further purification.

Conductive carbon black (Super P), polyvinylidene fluoride (PVDF), and N-methyl-2-pyrrolidone (NMP) were used for electrode fabrication. Lithium metal foil was used as the counter/reference electrode. The electrolyte consisted of 1 M LiPF_6_ dissolved in ethylene carbonate/dimethyl carbonate (EC/DEC, 3:7 *v*/*v*). Celgard 2300 membrane was employed as the separator.

Generative AI-assisted image generation was used solely for the preparation of schematic illustrations in this study. The conceptual design, scientific content, mechanistic interpretation, and all textual annotations were independently developed and verified by the authors based on experimental results and literature analysis. GPT Image 2.0 (OpenAI) was used to generate preliminary graphical elements according to author-defined visualization requirements, and the resulting images were subsequently manually edited, arranged, and finalized using Adobe Photoshop (version 2026). The authors reviewed and validated all figure contents and take full responsibility for the accuracy and integrity of the final published material.

### 2.2. Preparation of the FeS_2_/CuFeS_2_ Composites

**Figure 1:**
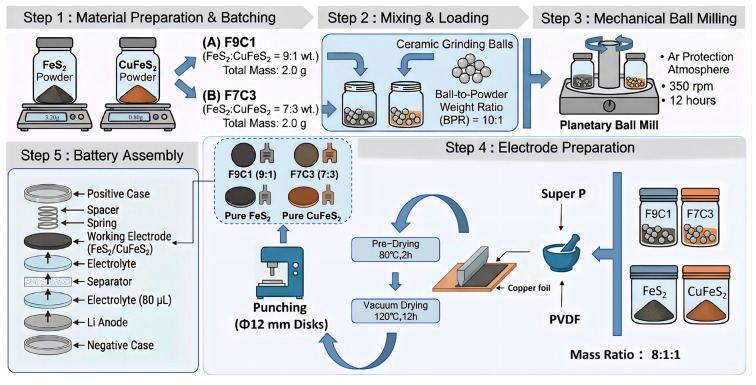
Synthesis route of FeS_2_/CuFeS_2_ composite material and negative electrode.

The FeS_2_/CuFeS_2_ composites were synthesized via a high-energy mechanochemical ball-milling method. Specifically, pristine FeS_2_ and CuFeS_2_ powders were precisely weighed and mixed at mass ratios of 9:1 and 7:3, maintaining a fixed total mass of 2.0 g for each batch. The mixed powders were transferred into a milling jar along with ceramic grinding balls (6 mm in diameter) at a constant ball-to-powder mass ratio of 10:1. After sealing under an argon atmosphere to minimize oxidation during milling, the jar was subjected to planetary ball milling (QM-3SP2, Nanjing Chishun, Nanjing, China) at 350 rpm for 12 h. This high-energy ball-milling process not only enables mixing but also induces particle size reduction, enhanced interfacial contact, and structural disorder, which can improve electrochemical kinetics compared to simple physical mixing. The obtained composite powders were collected in an argon-filled glovebox (MB-Unilab, M. Braun Inertgas Systems Co., Ltd., Shanghai, China) and directly used for subsequent electrode fabrication. The samples were denoted as F9C1 and F7C3, respectively. For comparison, pristine FeS_2_ and CuFeS_2_ powders were also directly fabricated into electrodes without any prior milling treatment The synthesis route of the FeS_2_/CuFeS_2_ composite material and the negative electrode is shown in Figure 1.

### 2.3. Electrode Preparation and Cell Assembly

The working electrodes were prepared by mixing the active material, conductive car bon black (Super P), and PVDF binder at a mass ratio of 8:1:1. N-methyl-2-pyrrolidone (NMP) was added as the solvent, and the mixture was thoroughly homogenized to form a uniform slurry. The slurry was coated onto copper foil using a doctor blade and initially dried at 80 °C for 2 h, followed by vacuum drying at 120 °C for 12 h to completely remove residual solvent. The dried electrodes were subsequently calendared to improve interparticle contact and punched into circular disks with a diameter of 12 mm. The active material mass loading was controlled within 1.0–1.3 mg cm^−2^ to ensure measurement consistency.

CR2032-type coin cells were assembled in an argon-filled glovebox with oxygen and moisture levels below 0.1 ppm. Lithium metal foil was used as the counter/reference electrode, while Celgard 2300 membrane served as the separator. Approximately 80 μL of electrolyte (1 M LiPF6 in EC/DEC, 3:7 *v*/*v*) was added to each cell. After assembly, the cells were aged at room temperature for 12 h before electrochemical measurements. We noticed that all reported specific capacities were calculated based solely on the mass of active materials, rather than the total electrode mass including conductive carbon and binder.

### 2.4. Structural Characterization

X-ray diffraction (XRD, Ultima IV, Rigaku Corporation, Tokyo, Japan) with Cu Kα radiation was employed to analyze the phase composition and crystal structure of the deep-sea polymetallic sulfide ore and the as-prepared FeS_2_/CuFeS_2_ composites. X-ray fluorescence (XRF, Axios mAX, Malvern Panalytical, Almelo, Netherlands) was used to determine the elemental composition and relative content of the ore sample. The microstructure, particle size, and agglomeration characteristics of the ore, single-phase precursors, and composite samples were examined by scanning electron microscopy (SEM, SU8010, Hitachi High-Tech Corporation, Tokyo, Japan), while the elemental distribution was analyzed by energy-dispersive X-ray spectroscopy (EDS).

### 2.5. Electrochemical Measurements

The electrochemical performance of the samples was evaluated using CR2032-type coin half-cells. Galvanostatic charge–discharge (GCD) measurements were carried out within a voltage range of 0.01–3.0 V (vs. Li^+^/Li) to evaluate the specific capacity, cycling stability, Coulombic efficiency, and rate capability of the electrodes.

Cyclic voltammetry (CV) measurements were performed on an electrochemical workstation at scan rates ranging from 0.1 to 1.0 mV s^−1^ to investigate the redox behavior and lithium-storage kinetics. Electrochemical impedance spectroscopy (EIS) measurements were performed over a frequency range from 100 kHz to 0.01 Hz with an AC amplitude of 5 mV at an initial potential of 3.0 V to investigate the charge-transfer behavior and interfacial reaction kinetics.

The galvanostatic intermittent titration technique (GITT) was employed to investigate Li^+^ diffusion kinetics. A constant current pulse of 112 μA was applied for 30 min, followed by a 30 min relaxation process under open-circuit conditions within a voltage window of 0.01–3.0 V. The lithium-ion diffusion coefficients were subsequently calculated based on the potential response during the pulse and relaxation processes.

## 3. Results and Discussion

This section commences with a comprehensive structural and compositional characterization of SMS ores using X-ray diffraction (XRD) and X-ray fluorescence (XRF), with the aim of elucidating their intrinsic mineralogical framework. Guided by these findings, FeS_2_ and CuFeS_2_—the two predominant sulfide phases—were selected as model systems for further investigation. Their fundamental morphological characteristics and intrinsic electrochemical behaviors were first examined in isolation. Subsequently, FeS_2_/CuFeS_2_ composites with controlled mass ratios were constructed via mechanochemical milling to emulate the natural mineralogical distribution.

### 3.1. Phase and Elemental Composition Analysis of SMS

To establish a foundational understanding of the mineralogical composition and its implications for electrode design, both pristine and ball-milled ore samples were systematically analyzed via XRD and XRF.

The XRD pattern of the pristine ore exhibits multiple sharp and well-resolved diffraction peaks, indicative of a highly crystalline multiphase system. Phase identification based on standard reference databases confirms the coexistence of pyrite (FeS_2_), chalcopyrite (CuFeS_2_), and sphalerite (ZnS), along with minor contributions from gangue minerals. Notably, FeS_2_ displays the highest peak density and intensity, signifying its dominance as the primary crystalline phase. In parallel, the characteristic reflections of CuFeS_2_ are clearly discernible, establishing it as a key copper-bearing active sulfide. Although ZnS is also detected, its comparatively weaker diffraction intensity suggests a subordinate contribution in terms of phase abundance or crystallographic prominence.

**Figure 2:**
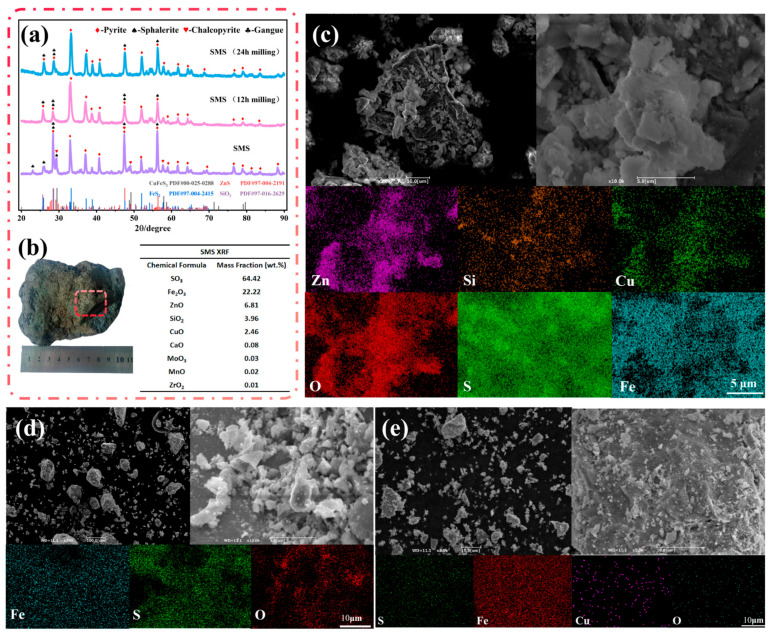
(**a**) XRD patterns of SMS ore before and after ball milling; (**b**) Photograph and XRF analysis of raw SMS ore; (**c**) SEM-EDS elemental mapping of milled SMS ore; (**d**) SEM-EDS of pure FeS_2_; (**e**) SEM-EDS of pure CuFeS_2_.

Complementary XRF analysis (Figure 2b) provides quantitative insight into the elemental distribution. The ore is characterized by a Fe–S dominated framework, accompanied by appreciable amounts of Cu and Zn, thereby constituting a typical multicomponent sulfide system. The high sulfur and iron content corroborates the predominance of iron sulfide phases, while the presence of copper confirms the existence of Cu–Fe–S active components. The detection of zinc further indicates the incorporation of Zn–S phases within the matrix. These elemental findings are in strong agreement with the multiphase features observed in XRD, collectively confirming that the ore represents a naturally evolved composite system centered on Fe–S chemistry, with secondary Cu–Fe–S and Zn–S phases.

The intrinsic multiphase nature of the pristine ore, as revealed by XRD, underscores the coexistence of diverse crystalline sulfides. When coupled with XRF results, it becomes evident that Fe and S dominate the compositional landscape, while Cu and Zn act as critical secondary elements contributing to functional diversity. Further phase analysis identifies FeS_2_ as the principal crystalline host, whereas CuFeS_2_ serves as a representative bimetallic sulfide phase with potential electrochemical activity.

Accordingly, FeS_2_ and CuFeS_2_ were rationally selected as the primary constituents for subsequent composite anode design. FeS_2_ represents the dominant Fe–S active matrix, while CuFeS_2_ embodies a secondary phase capable of introducing multimetallic synergy. Their integration not only faithfully reflects the intrinsic composition of natural deep-sea sulfide ores but also aligns with a rational design paradigm aimed at achieving a synergistic balance between high capacity and structural stability.

It should be noted that natural SMS is intrinsically a highly complex multicomponent mineral system, and the current study does not intend to fully replicate all mineral phases present in the original ore. Instead, this work should be regarded as a proof-of-concept exploration aimed at establishing the feasibility of constructing SMS-inspired composite sulfide anodes through mechanochemical processing. The incorporation of additional sulfide phases such as ZnS into more complex multicomponent model systems will be considered in future investigations.

### 3.2. Fundamental Characterization and Electrochemical Performance of Single-Phase FeS_2_ and CuFeS_2_

Building upon the aforementioned compositional analysis, chemically pure FeS_2_ and CuFeS_2_ were selected as model components for subsequent investigation. The as-purchased precursors exhibit high crystallinity and phase purity, rendering them suitable for composite construction and comparative analysis. Therefore, detailed crystallographic discussion is omitted here.

#### 3.2.1. Morphology and Elemental Distribution

To elucidate the intrinsic morphological characteristics of the single-phase precursors, scanning electron microscopy (SEM) and energy-dispersive X-ray spectroscopy (EDS) analyses were conducted. Both FeS_2_ and CuFeS_2_ powders exhibit irregular particulate morphologies with noticeable aggregation and agglomeration. FeS_2_ displays a relatively broad particle size distribution, characterized by the coexistence of bulk-like and fragmented particles. In contrast, CuFeS_2_ consists of finer particles with improved dispersion, occasionally revealing lamellar or fractured surfaces. EDS elemental mapping results show that Fe and S are relatively uniformly distributed in FeS_2_, while Cu, Fe, and S in CuFeS_2_ also exhibit no obvious elemental aggregation, indicating good compositional homogeneity in both materials.

Overall, both FeS_2_ and CuFeS_2_ possess the fundamental prerequisites for constructing composite electrode materials. FeS_2_ shows morphological characteristics typical of a dominant active component, whereas the relatively finer particle size and better dispersibility of CuFeS_2_ are expected to promote more intimate multiphase contact and enhanced interfacial coupling with FeS_2_ during the subsequent ball-milling process [32].

#### 3.2.2. Electrochemical Performance of Single-Phase Materials

**Figure 3:**
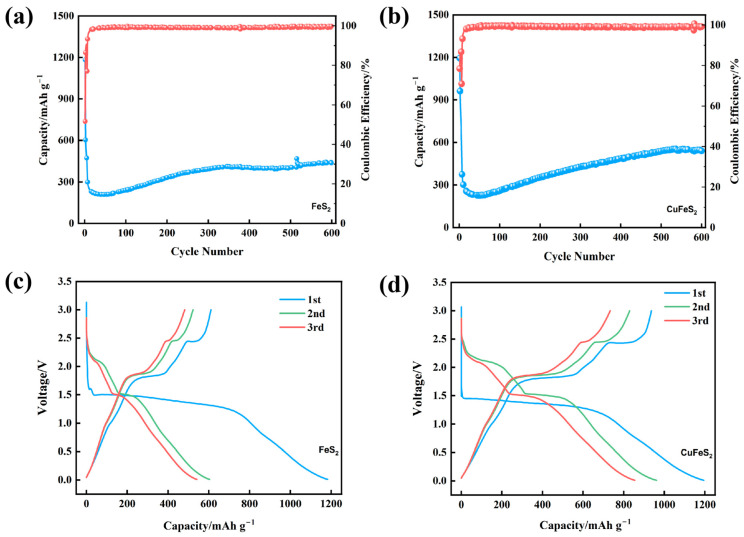
(**a**,**b**) Cycling stability and Coulombic efficiency of (**a**) FeS_2_ and (**b**) CuFeS_2_ electrodes; (**c**,**d**) Galvanostatic charge–discharge curves of (**c**) FeS_2_ and (**d**) CuFeS_2_ electrodes for the initial three cycles.

The cycling performance and Coulombic efficiency of FeS_2_ and CuFeS_2_ electrodes under identical testing conditions are presented in Figure 3a and Figure 3b, respectively.

Both electrodes deliver high initial discharge capacities, reaching 1182.6 and 1194.2 mAh g^−1^ for FeS_2_ and CuFeS_2_, respectively. These processes are mainly attributed to solid electrolyte interphase (SEI) formation, electrolyte decomposition, irreversible structural reconstruction, and partial trapping of lithium ions within defect-rich regions [33,34]. Similar electrochemical activation phenomena have been widely reported in conversion-type transition-metal sulfides, where repeated conversion reactions can induce progressive surface reconstruction, defect generation, and interfacial lithium-storage behavior during long-term cycling [35,36].

The relatively low initial Coulombic efficiencies (51.62% for FeS_2_ and 78.43% for CuFeS_2_) indicate substantial irreversible processes during the first lithiation cycle. These processes are mainly attributed to solid electrolyte interphase (SEI) formation, electrolyte decomposition, irreversible structural reconstruction, and partial trapping of lithium ions within defect-rich regions. Similar phenomena have been widely reported in conversion-type transition-metal sulfides, where electrochemically induced surface reconstruction and interfacial activation contribute significantly to the overall lithium-storage behavior.

The FeS_2_ electrode exhibits a characteristic “rapid decay–gradual recovery–stabilization” evolution. Following an initial discharge capacity approaching 1200 mAh g^−1^, the capacity sharply decreases to 604.1 mAh g^−1^ in the second cycle and further declines to approximately 298.3 mAh g^−1^ within the first several cycles. Such early-stage attenuation is mainly associated with severe volume variation, particle pulverization, unstable interfacial reactions, and partial loss of electrical contact during repeated conversion reactions.

Interestingly, prolonged cycling induces a gradual capacity recovery, with the reversible capacity stabilizing above 400 mAh g^−1^ after extended cycling. This activation phenomenon is likely related to continuous electrochemical reconstruction of the electrode microstructure during repeated lithiation/delithiation processes. The progressive fragmentation of particles can continuously expose fresh electrochemically active surfaces and generate additional defect sites, thereby enhancing pseudocapacitive lithium storage. Meanwhile, repeated conversion reactions may gradually improve the reversibility of Li_2_S decomposition and facilitate interfacial charge-transfer kinetics. In addition, reversible polymeric or gel-like surface layers generated from continuous electrolyte decomposition have also been proposed as a possible source of additional lithium-storage capacity during long-term cycling of conversion-type electrode materials [37].

Compared with FeS_2_, the CuFeS_2_ electrode exhibits a similar yet substantially improved electrochemical evolution behavior. Although an initial capacity decay is also observed, the subsequent recovery process is more pronounced and stable. After early-stage attenuation to ~245 mAh g^−1^ (23rd cycle), the reversible capacity gradually increases and eventually stabilizes at ~540 mAh g^−1^ after 600 cycles, significantly outperforming FeS_2_ under comparable conditions. The Coulombic efficiency also rapidly approaches ~100%, indicating improved reaction reversibility and interfacial stability.

The enhanced cycling stability and activation behavior of CuFeS_2_ can be attributed to its bimetallic sulfide framework and more favorable electrochemical reconstruction characteristics. The coexistence of Cu and Fe species may facilitate electron transport and accelerate the reversible conversion between metal sulfides and Li_2_S during cycling. Furthermore, the finer particle morphology and improved dispersion of CuFeS_2_ are expected to promote the formation of abundant interfacial active sites and shorten lithium-ion diffusion pathways, thereby enhancing pseudocapacitive contribution and long-term electrochemical reversibility [38,39].

Further insights from the galvanostatic charge–discharge profiles of the initial three cycles reveal pronounced irreversible features in both materials. Notably, CuFeS_2_ exhibits a higher degree of overlap between the second and third cycles, along with more well-defined voltage plateaus in subsequent cycles. This indicates that a stable and reversible reaction pathway is established more rapidly after the initial activation process. Such behavior is consistent with its superior long-term cycling performance, implying reduced polarization and enhanced structural retention.

Collectively, the combined morphological and electrochemical analyses reveal distinct yet complementary characteristics of the two sulfides. Both FeS_2_ and CuFeS_2_ possess high crystallinity and significant initial lithium storage activity. However, their cycling stability and reaction reversibility differ markedly. FeS_2_, with its broader particle size distribution and bulk-like morphology, delivers high initial capacity but suffers from severe early-stage degradation, followed by gradual electrochemical activation. In contrast, CuFeS_2_, with finer particles and better dispersion, demonstrates superior cycling stability, higher reversible capacity retention, and more stable electrochemical behavior [40,41].

These findings suggest a clear division of functional roles: FeS_2_ serves as a high-capacity active component, whereas CuFeS_2_ contributes structural stability and sustained electrochemical reversibility. Therefore, integrating these two phases is expected to yield a synergistic composite system that simultaneously achieves high specific capacity and enhanced structural durability. This insight provides a robust experimental and theoretical foundation for the rational design of FeS_2_/CuFeS_2_ composite anodes.

### 3.3. Morphology, Structure, and Electrochemical Performance of FeS_2_/CuFeS_2_ Composite Samples

#### 3.3.1. Morphology and Elemental Distribution of FeS_2_/CuFeS_2_ Composites

To emulate the tightly intergrown architecture of multiple mineral phases in SMS, FeS_2_/CuFeS_2_ composite systems with different mass ratios were fabricated via a mechanochemical route. Among them, the F9C1 composition (9:1) was designed to approximate the phase proportion of the natural SMS ore and was therefore selected as the principal target of this study, whereas F7C3 (7:3) was introduced as a comparative counterpart to evaluate the composition-dependent microstructural characteristics.

**Figure 4:**
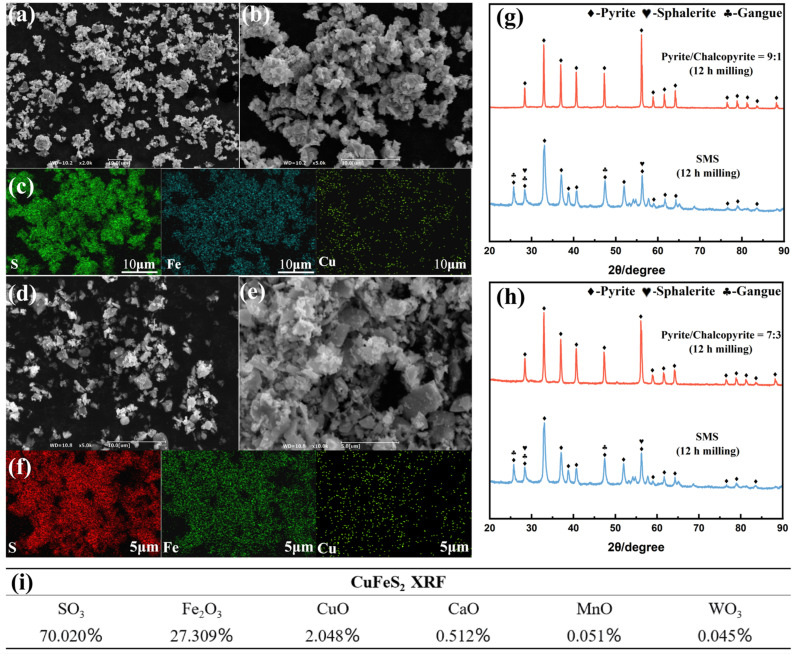
(**a**,**b**) SEM images of the F9C1 sample; (**c**) Corresponding EDS elemental mapping of S, Fe, and Cu for the F9C1 sample; (**d**,**e**) SEM images of the F7C3 sample; (**f**) Corresponding EDS elemental mapping of S, Fe, and Cu for the F7C3 sample; (**g**) XRD pattern of the F9C1 sample, with characteristic peaks indexed to FeS_2_; (**h**) XRD pattern of the F7C3 sample, with characteristic peaks indexed to FeS_2_. (**i**) XRF analysis results of the CuFeS_2_ component (wt.%).

The SEM images and corresponding EDS elemental maps of the ball-milled F9C1 and F7C3 samples are shown in Figure 4a–f. After mechanochemical treatment, both composites exhibit evident particle fracture, refinement, and agglomeration, while the boundaries of the original precursor particles become largely indistinguishable. This observation indicates that high-energy ball milling effectively promotes close contact and homogeneous mixing between the two sulfide phases. Overall, both samples are mainly composed of irregular block-like or clustered particles, suggesting that the milling process not only facilitates physical integration of the components but also induces substantial particle refinement and interfacial reconstruction [42,43].

Compared with F9C1, the F7C3 sample exhibits a relatively denser agglomerated structure, and the interparticle contacts appear tighter at higher magnification, implying enhanced multiphase interaction at higher CuFeS2 content. EDS elemental mapping further demonstrates that S, Fe, and Cu are distributed throughout the composite particles. The distributions of S and Fe remain relatively continuous across the observed region, whereas the Cu signal, although comparatively weaker, can still be detected without obvious localized enrichment. Such a distribution behavior is likely associated with the repeated impact and shear effects generated during high-energy milling, which promote uniform dispersion of CuFeS2 within the FeS2 matrix.

In the F9C1 sample, the elemental distribution remains dominated by Fe and S signals, while the Cu signal becomes noticeably weaker and more dispersed. This phenomenon mainly originates from the relatively low CuFeS_2_ content (10 wt.%) in the composite system. After ball-milling-induced homogenization, the local Cu concentration within a given observation area decreases substantially and may further approach the detection limitation of EDS analysis. Meanwhile, the strong FeS_2_ background may partially mask the Cu signal, resulting in a relatively diluted spatial distribution. Such behavior suggests that the F9C1 sample preserves a FeS_2_-dominated structural framework in which CuFeS_2_ exists as a uniformly dispersed minor phase.

Overall, mechanochemical milling proves effective for constructing FeS_2_/CuFeS_2_ composite structures with intimate multiphase contact and homogeneous elemental dispersion. F7C3 exhibits relatively stronger multiphase aggregation characteristics, whereas F9C1 more closely resembles a FeS_2_-dominated composite system containing finely dispersed Cu-bearing phases. From a biomimetic mineralization perspective, the resulting ball-milled microstructures—characterized by fine-particle agglomeration, multiphase intergrowth, and relatively uniform elemental distribution—show certain similarities to the tightly intergrown and finely disseminated textures commonly observed in natural SMS ores [44,45,46]. Therefore, mechanochemical processing can be regarded as an engineering strategy for reconstructing some key structural characteristics of natural sulfide mineral assemblages.

#### 3.3.2. Crystal Structure Analysis of FeS_2_/CuFeS_2_ Composites

The XRD patterns of the F9C1 and F7C3 composites are shown in Figure 4g,h. Both samples exhibit well-defined diffraction peaks, indicating that the materials retain a certain degree of crystallinity after ball milling. The main diffraction peaks are well aligned with the standard pattern of FeS_2_, confirming that FeS_2_ remains the dominant crystalline phase in the composite system.

A comparison of peak intensity and profile reveals noticeable structural differences between the two compositions. The F9C1 sample exhibits relatively stronger diffraction peaks, particularly near ~32.88° and ~56.12°, together with comparatively sharper peak profiles, suggesting that the crystalline framework of FeS_2_ is better preserved at lower CuFeS_2_ content. In contrast, the diffraction intensity of F7C3 decreases to some extent, indicating that increasing the CuFeS_2_ proportion may enhance particle refinement and structural disorder during high-energy milling. This observation is also consistent with the SEM results, demonstrating that mechanochemical treatment promotes close integration between different sulfide phases while simultaneously introducing non-equilibrium structural features.

Notably, no distinct diffraction peaks corresponding to CuFeS_2_ are observed in either composite sample. In addition, XRF analysis of the purchased CuFeS_2_ precursor confirmed the presence of Cu-containing components, with characteristic CuO, Fe_2_O_3_, and SO_3_ signals detected in the raw material (Figure 4i). Combined with the EDS elemental mapping results, the XRF data further support the successful incorporation and dispersion of Cu-bearing phases within the composite system, although their crystalline diffraction features become difficult to distinguish after intensive mechanochemical treatment. This does not imply the absence of CuFeS_2_, but rather suggests significant structural evolution during the ball-milling process. As evidenced by the XRD analysis of the raw SMS ore, clear CuFeS_2_ diffraction peaks are present in the pristine sample, whereas they become markedly weakened or even disappear after 12 h and 24 h of ball milling.

From a structural perspective, continuous impact and shear forces generated during ball milling can lead to substantial grain refinement, lattice distortion, and reduced crystallinity. In addition, the relatively low CuFeS_2_ content, particularly in F9C1, further weakens its diffraction contribution, which can be easily obscured by the dominant FeS_2_ phase and overlapping sulfide diffraction signals. Therefore, the absence of identifiable CuFeS2 diffraction peaks should not be directly interpreted as the disappearance of Cu-containing phases, but rather as a consequence of reduced crystallinity and limited phase detectability after high-energy milling.

Consequently, it is reasonable to infer that CuFeS_2_ in the present FeS_2_/CuFeS_2_ composite system likely exists in a highly dispersed and low-crystallinity state rather than as a well-defined independent crystalline phase. Such a structural feature resembles the fine-grained intergrowth and closely associated multiphase characteristics commonly observed in natural SMS, and may contribute to the formation of abundant heterogeneous interfaces within the composite system.

#### 3.3.3. Cycling Performance of FeS_2_/CuFeS_2_ Composites

**Figure 5:**
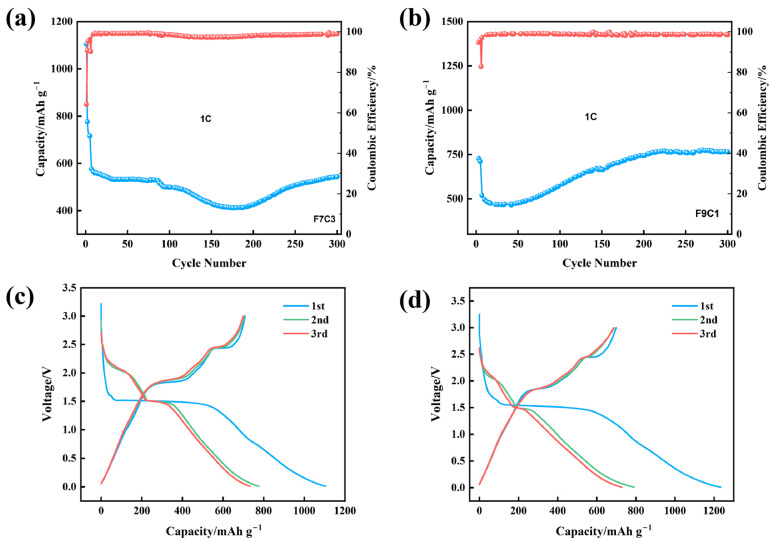
(**a**,**b**) Cycling stability and Coulombic efficiency of (**a**) F7C3 and (**b**) F9C1 electrodes at 1 C; (**c**,**d**) Galvanostatic charge–discharge curves of (**c**) F7C3 and (**d**) F9C1 electrodes for the initial three cycles.

To evaluate the long-term lithium-storage performance of the FeS_2_/CuFeS_2_ composites with different phase ratios, cycling tests were conducted at 1 C for both F7C3 and F9C1. The cycling performance and corresponding Coulombic efficiency curves are presented in Figure 5a,b. Both electrodes exhibit high initial discharge capacities and rapidly increasing Coulombic efficiencies during the initial cycles, eventually stabilizing near 100%, indicating progressively improved electrochemical reversibility after the initial activation process.

For F7C3, the first-cycle discharge capacity reaches 1104.7 mAh g^−1^, while the initial Coulombic efficiency is only 64.16%, suggesting substantial irreversible lithium consumption during the first lithiation process. The reversible capacity subsequently decreases rapidly to 576.4 mAh g^−1^ by the 7th cycle and then gradually decays during prolonged cycling. A minimum capacity of 423.2 mAh g^−1^ is observed near the 155th cycle, followed by a moderate recovery to 543.3 mAh g^−1^ after 300 cycles.

In contrast, F9C1 exhibits significantly improved cycling behavior. At the third cycle, where stable cycling begins, the discharge capacity already reaches approximately 726.9 mAh g^−1^, accompanied by a Coulombic efficiency of 94.76%, indicating faster interfacial stabilization during the early stage. Although the capacity initially decreases to approximately 475 mAh g^−1^ at the 16th cycle, a sustained activation-induced recovery is subsequently observed. The reversible capacity gradually increases to 743.5 mAh g^−1^ after approximately 200 cycles and further reaches 763.4 mAh g^−1^ after 300 cycles, substantially higher than that of F7C3 under identical conditions.

Such gradual capacity recovery during prolonged cycling has been widely reported in conversion-type transition-metal sulfide electrodes and is generally associated with continuous electrochemical activation and structural evolution during repeated lithiation/delithiation reactions. Previous studies have shown that repeated conversion reactions may induce progressive particle refinement, local structural disorder, and conversion-induced nanostructuring, thereby generating additional electrochemically active interfaces and shortening ion/electron transport pathways [2,21]. Meanwhile, repeated volume variation during cycling may continuously expose fresh active surfaces and defect-rich regions, which can further contribute to interfacial lithium-storage activity.

In addition, progressively enhanced pseudocapacitive contribution during long-term cycling has been suggested to partially account for the activation behavior observed in transition-metal sulfides, owing to the gradual evolution of electrochemically active interfaces and surface-controlled storage processes [21]. As further supported by the kinetic analysis in Section 3.3.7, F9C1 exhibits substantial surface-controlled pseudocapacitive behavior, particularly at high scan rates. The gradual enhancement of pseudocapacitive lithium storage during cycling may therefore partially account for the sustained late-stage capacity increase observed for F9C1.

Furthermore, previous studies have proposed that reversible polymeric or gel-like surface films generated from electrolyte decomposition may also provide additional lithium-storage contribution during long-term cycling of conversion-type electrodes [47]. Therefore, the activation behavior observed in the present work is likely associated with the combined effects of electrochemical reconstruction, interfacial activation, surface-controlled lithium storage, and gradual evolution of electrode microstructure during repeated cycling.

Compared with F7C3, the superior cycling stability and activation behavior of F9C1 may originate from its more balanced FeS_2_/CuFeS_2_ phase ratio, which is favorable for maintaining relatively stable electrochemical evolution during prolonged cycling. However, because no post-cycling structural characterization was conducted in the present work, the detailed structural evolution mechanism after extended cycling still requires further investigation.

Overall, both composites display characteristic activation behavior during prolonged cycling, whereas F9C1 demonstrates clearly superior capacity retention, reversibility, and late-stage capacity recovery.

#### 3.3.4. Galvanostatic Charge–Discharge Behavior of FeS_2_/CuFeS_2_ Composites

The galvanostatic charge–discharge profiles of the first three cycles for F7C3 and F9C1 are shown in Figure 5c,d. Both electrodes exhibit pronounced first-cycle irreversibility, where the initial discharge capacities are substantially higher than those of subsequent cycles. This behavior is commonly observed in conversion-type sulfide electrodes and is mainly associated with SEI formation and irreversible side reactions during the initial lithiation process [47,48,49]. For both samples, a distinct discharge plateau centered near 1.5 V is observed, corresponding to the characteristic conversion reactions of FeS_2_- and CuFeS_2_-based sulfides with lithium ions. However, clear differences are observed in the subsequent cycling behavior of the two electrodes.

For F7C3, the first discharge curve differs substantially from those of the subsequent cycles. Quantitatively, at a discharge capacity of 50 mAh g^−1^, the first-cycle discharge and charge voltages are 2.1482 and 1.7618 V, respectively, corresponding to a polarization gap of 0.3864 V. Even by the third cycle, the voltage gap at the same capacity remains as high as 0.1791 V. In terms of curve overlap, at a discharge capacity of 20 mAh g^−1^, the second- and third-cycle discharge voltages are 2.1385 and 2.1596 V, respectively, with a difference of 0.0211 V. These results suggest that appreciable kinetic hindrance and polarization persist during subsequent cycling.

By contrast, F9C1 exhibits markedly improved overlap between the second and third cycles. At the same discharge capacity of 50 mAh g^−1^, the first-cycle polarization gap is only 0.1925 V, substantially smaller than that of F7C3. By the third cycle, this value decreases further to only 0.0562 V, indicating effective mitigation of polarization. Likewise, at a discharge capacity of 20 mAh g^−1^, the second- and third-cycle discharge voltages are 2.1106 and 2.1168 V, respectively, with a minimal difference of only 0.0062 V, far lower than that of F7C3. The voltage plateaus in subsequent cycles also remain clearer and more stable, suggesting that F9C1 establishes a reversible reaction pathway more rapidly after the initial activation process, with superior reaction kinetics and cycling stability. In addition, after prolonged cycling, the discharge cutoff voltage of F7C3 decreases to 2.4445 V at the 300th cycle, whereas F9C1 still maintains 2.5347 V, suggesting comparatively reduced polarization accumulation and more stable electrochemical evolution during long-term cycling. Combined with the cycling results, these observations indicate that F9C1 is able to maintain a more reversible electrochemical reaction process after repeated activation.

It should also be noted that the relatively low initial Coulombic efficiency observed in the present work is a common characteristic of conversion-type sulfide anodes, mainly originating from extensive SEI formation, irreversible electrolyte decomposition, and structural reconstruction during the first lithiation process. Although such irreversible lithium consumption may limit practical full-cell energy density, the present study primarily aims to investigate the fundamental electrochemical behavior and mechanochemical synergistic effects of SMS-inspired sulfide composite systems in half-cell configurations. Further optimization strategies, including prelithiation engineering, electrolyte regulation, and surface/interface modification, may be necessary for future practical full-cell applications.

#### 3.3.5. Rate Capability of FeS_2_/CuFeS_2_ Composites

**Figure 6:**
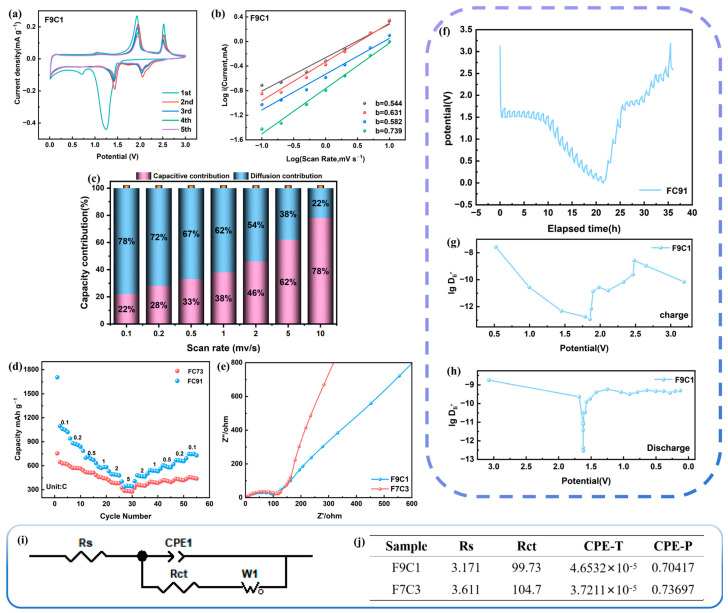
(**a**) CV curves of F9C1 at 0.1 mV s^−1^; (**b**) log(i) vs. log(v) for representative redox peaks; (**c**) Capacitive vs. diffusion-controlled contributions at various scan rates; (**d**) Rate performance of F7C3 and F9C1 at different current densities; (**e**) EIS Nyquist plots of F7C3 and F9C1; (**f**) Long-term potential-time curve of F9C1; (**g**,**h**) Li^+^ diffusion coefficients vs. potential during charge and discharge (GITT); (**i**) Equivalent circuit model used for EIS fitting; (**j**) Fitted electrochemical impedance parameters of F9C1 and F7C3.

The rate performance comparison between F7C3 and F9C1 is shown in Figure 6d. As the current density increases stepwise from 0.1 C to 0.2 C, 0.5 C, 1 C, 2 C, and 5 C, the specific capacities of both electrodes decrease to varying degrees, indicating that reaction kinetics become progressively constrained under high-rate conditions. Nevertheless, F9C1 consistently maintains higher reversible capacities than F7C3 at all tested rates, demonstrating clearly superior rate capability.

Using the stabilized capacity at 0.1 C as the reference, F9C1 retains approximately 73% of its baseline capacity at 1 C, whereas F7C3 retains only 58%. When the current density is further increased to 5 C, F9C1 still preserves 46% of its reference capacity, while F7C3 declines to only 34%. More importantly, after exposure to the 5 C high-rate regime and subsequent recovery to 0.1 C, the capacity of F9C1 rapidly returns to its initial level. Indeed, its reversible capacity during the following 2 C cycling even exceeds that recorded in the first round at the same rate, suggesting that high-rate cycling not only fails to damage the structure but may further promote electrode activation. By comparison, F7C3 suffers more severe capacity loss at high rates and exhibits weaker recovery ability. Although its initial capacity is relatively high, the pronounced fading observed during cycling indicates inferior electronic transport, ion diffusion, and structural robustness relative to F9C1.

These results further demonstrate that the optimized 9:1 composition enables improved electrochemical kinetics and better capacity retention under high-rate conditions.

#### 3.3.6. Cyclic Voltammetry Analysis of the F9C1 Electrode

To further investigate the electrochemical reaction behavior of the optimized F9C1 electrode, cyclic voltammetry (CV) measurements were conducted within the voltage range of 0–3 V, as shown in Figure 6a. During the initial cathodic scan, a pronounced reduction peak centered near 1.34 V is observed, which is mainly associated with the conversion reactions of FeS_2_/CuFeS_2_ during lithiation, accompanied by the formation of the solid electrolyte interphase (SEI) layer and irreversible electrolyte decomposition. In the subsequent anodic process, two distinct oxidation peaks located near 2.01 and 2.52 V can be identified, corresponding to the reversible oxidation of metallic Fe/Cu species and Li_2_S generated during the discharge process.

Notably, from the second cycle onward, the CV curves gradually overlap with each other, while both the peak positions and peak shapes remain highly consistent during subsequent scans. Such behavior suggests that after the initial electrochemical activation process, the internal reaction pathway of the electrode becomes progressively stabilized, accompanied by reduced polarization and improved reversibility. The high degree of overlap among the later CV curves further indicates relatively stable electrode/electrolyte interfaces and favorable structural stability during repeated lithiation/delithiation processes.

The evolution of the CV profiles also implies that electrochemical reconstruction may occur during early cycling. Similar activation behavior has been widely reported in conversion-type transition-metal sulfide anodes, where repeated conversion reactions can induce gradual nanostructuring, interfacial reconstruction, and increased electrochemically active surface area, thereby contributing to improved lithium-storage kinetics during prolonged cycling [50,51].

#### 3.3.7. Lithium-Storage Kinetics and Pseudocapacitive Contribution of F9C1

To further elucidate the lithium-storage kinetics of F9C1, CV measurements at different scan rates were analyzed according to the power-law relationship between peak current (***i***) and scan rate (***v***):

$$i=av^{b}$$

where ***a*** and ***b*** are adjustable parameters. In general, ***a b***-value of 0.5 indicates a diffusion-controlled process, whereas ***a b***-value approaching 1.0 suggests a surface-controlled capacitive process. Based on the linear fitting of log(***i***) versus log(***v***) for the representative redox peaks (Figure 6b), the calculated ***b***-values are 0.544, 0.631, 0.582, and 0.739, respectively. Since all values fall between 0.5 and 1.0, the lithium-storage behavior of F9C1 is governed by the synergistic contribution of diffusion-controlled conversion reactions and surface-controlled pseudocapacitive processes. Notably, the relatively high ***b***-value (~0.739) indicates a substantial pseudocapacitive contribution at certain electrochemical stages, which is beneficial for rapid charge transfer and enhanced rate capability [52,53].

To quantitatively distinguish the capacitive and diffusion-controlled contributions, the classical Dunn method was further employed according to the following relationship [53]:

$$i\left(V\right)=k_{1}v+k_{2}v^{1/2}$$

where $k_{1}v$ corresponds to the capacitive contribution and $k_{2}v^{1/2}$ represents the diffusion-controlled contribution. The calculated results (Figure 6c) reveal that the pseudocapacitive contribution gradually increases with increasing scan rate. Specifically, the capacitive contributions at 0.1, 0.2, 0.5, 1, 2, 5, and 10 mV s^−1^ are approximately 22%, 28%, 33%, 38%, 46%, 62%, and 78%, respectively. while the diffusion-controlled fractions decrease correspondingly from 78% to 22%. The gradual increase in pseudocapacitive contribution with increasing scan rate indicates that surface-controlled charge storage progressively dominates under high-rate conditions, thereby facilitating fast electrochemical response and improved rate capability [54].

It should be noted that these pseudocapacitive contributions were measured using thin electrodes with a relatively low mass loading (~1.0–1.3 mg cm^−2^). In practical thick-electrode systems with higher areal loading, ion diffusion limitations may reduce the pseudocapacitive contribution.

In addition, the substantial pseudocapacitive contribution may also partially account for the long-term activation behavior observed during cycling. Continuous conversion reactions and electrochemical reconstruction can progressively generate additional electrochemically active interfaces, defect sites, and nanoscale domains, thereby enhancing surface-controlled lithium storage during prolonged cycling.

Taken together, the combined b-value analysis and Dunn-method quantification demonstrate that F9C1 integrates both diffusion-controlled and capacitive lithium-storage mechanisms, while exhibiting a pronounced pseudocapacitive advantage under high-rate conditions.

#### 3.3.8. Electrochemical Impedance and GITT Diffusion Kinetics

The electrochemical impedance spectra (EIS) of F9C1 and F7C3 are shown in Figure 6e. Both electrodes exhibit a depressed semicircle in the high-frequency region followed by an inclined straight line in the low-frequency region, corresponding to the interfacial charge-transfer process and lithium-ion diffusion behavior, respectively. To further analyze the impedance characteristics, the spectra were fitted using the equivalent circuit shown in Figure 6i, consisting of the solution resistance (Rs), charge-transfer resistance (Rct), constant phase element (CPE), and Warburg diffusion element (W).

The fitted parameters are summarized in Figure 6j. The Rs values of F9C1 and F7C3 are 3.171 and 3.611 Ω, respectively, indicating similar electrolyte and contact resistance in both cells. More importantly, the Rct value of F9C1 (99.73 Ω) is slightly lower than that of F7C3 (104.7 Ω), suggesting relatively improved interfacial charge-transfer kinetics for the optimized 9:1 composition. In addition, F9C1 exhibits a more favorable low-frequency diffusion response, indicating enhanced Li^+^ transport behavior within the electrode.

To further investigate the ion-transport kinetics of the optimized F9C1 electrode, galvanostatic intermittent titration technique (GITT) measurements were performed, and the corresponding results are shown in Figure 6f–h. During the GITT test, a constant current pulse of 112 μA was applied for 30 min, followed by a 30 min relaxation process. The Li^+^ diffusion coefficients were calculated according to the following equation:

$$D^{GITT}=\frac{4}{\pi \tau }{\left(\frac{m_{B}V_{M}}{M_{B}S}\right)}^{2}{\left(\frac{{∆E}_{s}}{{∆E}_{t}}\right)}^{2}$$

where τ is the duration of the current pulse, $m_{B}$ is the mass of the active material, $V_{M}$ is the molar volume, $M_{B}$ is the molar mass, ***S*** is the electrode/electrolyte contact area, ${∆E}_{S}$ is the steady-state voltage change after relaxation, and ${∆E}_{t}$ is the transient voltage change during the current pulse after excluding the IR drop. The calculation was conducted based on the assumption of semi-infinite diffusion.

The GITT potential profile exhibits a characteristic stepwise response during both discharge and charge processes. Upon current application, the cell potential changes rapidly and subsequently relaxes gradually toward equilibrium during the open-circuit period, reflecting the coexistence of electrochemical polarization and diffusion relaxation processes. During discharge, a pronounced voltage plateau appears around 1.5–1.7 V, corresponding to the principal conversion-reaction region of the sulfide electrode.

The calculated Li^+^ diffusion coefficients exhibit strong potential dependence throughout the electrochemical process. In the high-potential region, the lg D Li^+^ values remain relatively high (~10^−8^–10^−9^ cm^2^ s^−1^). However, as the potential approaches the major conversion plateau, the diffusion coefficient decreases sharply to approximately 10^−13^ cm^2^ s^−1^. This pronounced decrease may be associated with the intensive conversion reactions occurring in this voltage region, where the formation of Li_2_S and metallic Fe/Cu phases induces substantial phase reconstruction and temporarily suppresses Li^+^ diffusion kinetics. Similar low-diffusion regions near conversion plateaus have also been reported in other transition-metal sulfide anodes.

After completion of the major conversion reaction, the diffusion coefficient rapidly recovers in the low-potential region, suggesting that the newly generated nanostructured conversion products provide shortened diffusion pathways and facilitate subsequent Li^+^ transport. A similar trend is also observed during charging, where the diffusion coefficients are generally slightly higher than those during discharge, indicating relatively improved delithiation kinetics.

Combined with the CV and rate-performance analyses, the EIS and GITT results consistently demonstrate that the optimized 9:1 FeS_2_/CuFeS_2_ composition enables improved coupled electron/ion transport kinetics and contributes to the superior electrochemical performance of F9C1.

#### 3.3.9. Discussion of Composition-Dependent Effects and the Structure–Performance Relationship

Based on the above structural, morphological, and electrochemical analyses, the FeS_2_/CuFeS_2_ composite system exhibits a distinct dependence of electrochemical behavior on phase composition, particularly when comparing the F7C3 and F9C1 samples.

From the structural characterization, both composites retain identifiable crystalline features after mechanochemical processing, with FeS_2_ remaining the dominant phase. However, the XRD results reveal that F9C1 exhibits relatively stronger and sharper diffraction peaks, suggesting that the FeS_2_ crystalline framework is better preserved at lower CuFeS_2_ content. In contrast, the reduced diffraction intensity observed in F7C3 indicates a higher degree of structural disorder, which is consistent with the enhanced particle refinement and more intensive mechanical interaction inferred from SEM observations.

At the microstructural level, both F7C3 and F9C1 show significant particle fragmentation, agglomeration, and intimate multiphase contact after ball milling. EDS mapping confirms that Cu-containing species are distributed throughout the composites without obvious segregation. Nevertheless, clear differences can be identified between the two compositions. F9C1 maintains a FeS_2_-dominated framework with finely dispersed Cu-bearing phases, whereas F7C3 exhibits a relatively denser agglomerated structure with stronger multiphase intergrowth. Such differences suggest that the relative proportion of CuFeS_2_ plays an important role in regulating the extent of multiphase interaction and structural compactness.

These structural and microstructural features are closely correlated with the electrochemical behavior. In cycling performance tests, F9C1 demonstrates significantly higher reversible capacity and more pronounced activation-induced capacity recovery compared with F7C3. After prolonged cycling, F9C1 achieves a reversible capacity exceeding 760 mAh g^−1^, whereas F7C3 stabilizes at a lower level. The Coulombic efficiency of F9C1 also increases more rapidly during early cycles, indicating faster establishment of a stable electrode/electrolyte interface.

The galvanostatic charge–discharge profiles further highlight the difference in reaction reversibility. F9C1 exhibits smaller polarization gaps and a higher degree of overlap between subsequent cycles, indicating reduced kinetic hindrance and improved reaction stability after the initial activation process. In contrast, F7C3 maintains relatively larger polarization and less consistent curve overlap, suggesting slower stabilization of the electrochemical reaction pathway.

The rate performance results provide additional evidence of composition-dependent kinetics. F9C1 consistently delivers higher capacities than F7C3 across all tested current densities and shows better capacity retention at high rates. Moreover, its capacity can be effectively recovered after high-rate cycling, implying more robust structural stability and faster reaction kinetics.

Kinetic analyses further support these observations. The CV results of F9C1 indicate a combination of diffusion-controlled and pseudocapacitive processes, with a progressively increasing capacitive contribution at higher scan rates. EIS measurements show that F9C1 possesses a slightly lower charge-transfer resistance than F7C3, while GITT analysis reveals relatively favorable Li^+^ diffusion behavior outside the main conversion plateau region. These results collectively suggest that the optimized composition facilitates more efficient electron/ion transport and interfacial reaction processes.

Combining the above findings, the improved electrochemical performance of F9C1 can be attributed to the synergistic effect of its structural configuration and phase distribution. The FeS_2_-dominated framework provides the primary capacity contribution, while the incorporation of a moderate amount of CuFeS_2_ promotes multiphase interaction, enhances interfacial activity, and improves reaction kinetics. When the CuFeS_2_ content is increased, as in F7C3, the resulting structural disorder and intensified agglomeration may partially hinder charge transport and reduce the overall electrochemical efficiency.

**Figure 7:**
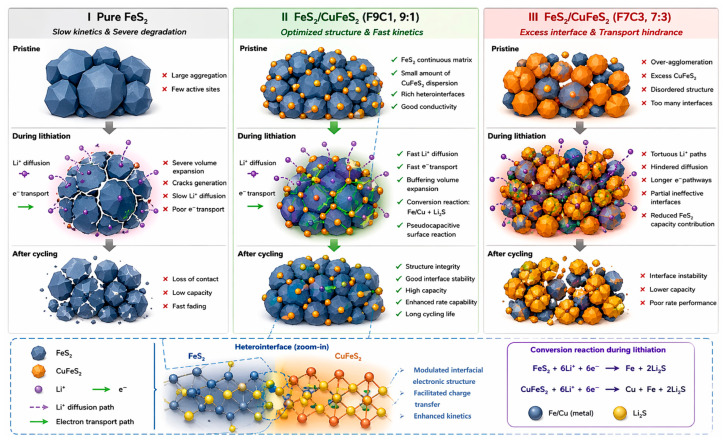
Proposed lithium storage mechanism comparison of pure FeS2, F9C1, and F7C3 electrodes.

Overall, the present results indicate that an appropriate balance between the dominant FeS_2_ phase and the secondary CuFeS_2_ phase is essential for achieving favorable electrochemical performance. The optimized F9C1 composition demonstrates that controlled multiphase coupling, rather than simple compositional increase in secondary components, plays a key role in regulating the structure–performance relationship in SMS-inspired sulfide composite anodes. A comparison of the proposed lithium storage mechanisms for the pure FeS_2_, F9C1, and F7C3 electrodes is presented in Figure 7.

## 4. Conclusions

In this work, FeS_2_/CuFeS_2_ composite anodes with different compositional ratios were constructed through a mechanochemical strategy inspired by the dominant sulfide phases in seafloor massive sulfides (SMS). By systematically comparing the electrochemical behavior and structural characteristics of F9C1 and F7C3, the following conclusions can be drawn:

(1) The FeS_2_/CuFeS_2_ phase ratio plays a critical role in regulating the structural and electrochemical behavior of the composite system. The F9C1 composition (9:1) preserves the FeS_2_-dominated crystalline framework more effectively while introducing finely dispersed Cu-containing phases that contribute to interfacial regulation. In contrast, excessive CuFeS_2_ incorporation in F7C3 leads to stronger agglomeration and increased structural disorder after mechanochemical treatment.

(2) The optimized F9C1 electrode exhibits superior electrochemical performance compared with F7C3, delivering a reversible capacity of 763.4 mAh g^−1^ after 300 cycles at 1 C together with improved rate capability and reduced polarization evolution during cycling. The results suggest that rational compositional optimization can effectively balance capacity contribution and structural stability in conversion-type sulfide anodes.

(3) Electrochemical kinetic analyses indicate that F9C1 possesses lower charge-transfer resistance, improved Li^+^ diffusion behavior, and substantial pseudocapacitive contribution. The gradual activation behavior observed during prolonged cycling is likely associated with the combined effects of electrochemical reconstruction, interfacial evolution, and enhanced surface-controlled lithium-storage processes. These findings suggest that introducing an appropriate amount of CuFeS_2_ may help regulate coupled electron/ion transport and interfacial electrochemical behavior within the FeS_2_ matrix.

(4) More broadly, this work demonstrates that compositionally inspired multiphase sulfide design can provide an effective route for improving the electrochemical performance of FeS_2_-based conversion-type anodes. Rather than directly reproducing natural mineralization processes, the present mechanochemical strategy should be regarded as an engineering analogue that reconstructs certain structural characteristics of naturally intergrown sulfide systems. The results provide useful insight into composition-dependent optimization of multiphase sulfide electrodes and may offer guidance for the future design of low-cost transition-metal sulfide anodes.

It should also be noted that the present study mainly focuses on fundamental half-cell electrochemical behavior at relatively low mass loading. Further investigations involving practical full-cell configurations, high-loading electrodes, long-term structural evolution, and post-cycling characterization will be necessary to fully evaluate the practical applicability of the proposed composite system.

## Figures and Tables

**Table 1:** Comparison of electrochemical performance between reported FeS_2_-based anodes and the present work.

Material	Current Density	Capacity Retention	Cycle Number	ICE	Ref.
FeS_2_/rGO	580 mAh g^−1^	100	0.1 A g^−1^	63%	Zhou et al. [10]
FeS_2_/CoS/C	710 mAh g^−1^	200	1 A g^−1^	—	Liu et al. [11]
CuFeS_2_/EG	481.6 mAh g^−1^	300	0.5 A g^−1^	—	Li et al. [20]
FeS_2_ nanospheres	541.5 mAh g^−1^	100	1 A g^−1^	51.62%	Wang et al. [21]
F9C1 (This work)	763.4 mAh g^−1^	300	1 C	64.16%	This work

## Data Availability

The original contributions presented in this study are included in the article. Further inquiries can be directed to the corresponding author.
